# William Cullen: A Psychological Study

**Published:** 1860-01-01

**Authors:** 


### Art. V.—WILLIAM CULLEN:—A PSYCHOLOGICAL STUDY.*

William Cullen was born at Hamilton, in Scotland, on the 15th of April, 1710. Of his boyhood little is known, save that he was a lad of lively manner, uncommon quickness of apprehension, and great retentiveness of memory. From this text the psychologist seeks to ascertain in what manner grew an intellectual superiority which placed its possessor among the giants of medical science, and in what that intellectual superiority chiefly consisted. The substratum, so far as known, cannot be said to be a rare one; for there are few schools in which could not be found lads whose disposition, readiness of apprehension, and power of memory might not be described in terms similar to those which have been used to distinguish Cullen's boyish characteristics. Hence it may be that, in tracing the development of Cullen's mind, we may learn in some degree why it is that Cullens are so few, while the species of soil from which he grew is plentiful.

Cullen received his preliminary tuition in the Grammar School of Hamilton, at the hands of a teacher of much repute. That this instruction was solid and substantial is certain from the circumstances under which it was given; that Cullen benefited by it to the full is evinced by the opening of his medical studies. From the Hamilton Grammar School he was sent to the University of Glasgow. We know little of the character of his studies in that university, except that his name is to be found in the list of students who, in 1727, attended the mathematical lectures of the celebrated Dr. Simson. The principal facts of this period of Cullen's life are these:—He was bound an apprentice to a Mr. John Paisley, a member of the Faculty of Physicians and Surgeons at Glasgow, and in extensive practice in that city, and

* "An Account of the Life, Lectures, and Writings of William Cullen, M.D., Professor of the practice of Physic in the University of Edinburgh. Commenced by Dr. John Thomson and Dr. William Thomson, and concluded by David Cragie, M.D." Edinburgh: Blackwood and Sons. 1859.

while with that gentleman he had an advantage which may be fairly presumed to have been a chief fostering cause of Cullen's future greatness.

Mr. Paisley was a man of studious disposition, and he had collected a large and valuable medical library. To this Cullen had free access. He had received an education fitting him to comprehend what he read; he had a master who knew the value of reading; he had books—at that time a rare advantage—and so prepared and so guided, he early cultivated a taste for reading. Thus, while he listened to professorial lectures, while he was enabled to mark the practical working of the medical art, he had at his command the teachings of the great masters of that art. That this latter advantage was the differential element which determined the active habits of inquiry that marked the whole of his life, may be assumed from the literary characteristics of that life, and from the value which he attached to the use of a library by students during his professorial career, as shown by the following incidents.

When Cullen, many years after his apprenticeship, became a lecturer in the University of Glasgow, the library of Mr. Paisley, as a mark of regard to Cullen, was thrown open to all his students; and, subsequently, in Edinburgh, Cullen gave to his class the use of his library.

"Home-keeping youths have ever homely wits," is as true of the student who has read little as of the untravelled. Books are the world of the student, and he who has not made good use of them while a student will be apt to turn out a mere academical mental instrument. It may be a highly polished and well perfected one, but one which the possessor knows not how to use, or sees the necessity for using. For if he lack a wider acquaintance with those giants of humanity whose thoughts govern the world's mind, than what is contemplated in an academical course, he will be apt to lack the means wherewithal truly to gauge his own knowledge, and will fail of those great mind-born incentives to research and labour which stir up the thoughts of man from the foundation. He will be apt, indeed, to look upon his academical career as the highest aim of his intellectual life, and not the means to a higher life; to regard as the culmination of his mental training what should be simply the foundation of his own self-dependent efforts of research. In Mr. Paisley's library we deem that Cullen laid the groundwork of his after fame.

"—— Blest
Is he whose heart is the home of the great dead,
And their great thoughts."

During his student life Cullen was noted for great diligence, and an active observation. If, when in conversation with his fellow-students, any subject was introduced with which he was imperfectly acquainted, he would take little share in the conversation; but if, at some future period, the subject were again broached, he would show that in the meantime he had gained an accurate familiarity with it in all its details. A disposition of this kind lets few things escape it; hence we are not surprised to find during the course of Cullen's life, that he manifested an intimate knowledge of many subjects.

Very early in his medical career he had directed his attention particularly to the subject of Materia Medica, and when studying at the University of Edinburgh, he, along with other students of congenial habits, formed a society for mutual improvement, which proved the foundation of the Medical Society of that city. After leaving Edinburgh and commencing the practice of his profession at Hamilton, he devoted considerable attention to the study of general literature and philosophy, and about the same period, "being much addicted to study," as he himself says, he began to review the system of medicine then taught in Scotland. Indeed, during his several years' residence at Hamilton, he devoted himself unremittingly to a critical study of medicine and the collateral sciences, as well as to the actual practice of his profession; and there first were developed those medical notions, as well as that exact acquaintance with disease, which many years afterwards caused his name to become so celebrated as a philosophical physician, and a teacher. At this period of his life, he pursued his researches in physical science experimentally as well as theoretically, and he collected a library containing a number of the most rare and valuable books on medicine. After this probation of severe study and observation, he removed to Glasgow, and very shortly after taking up his residence there, he commenced lecturing on the Theory and Practice of Medicine in the University; and subsequently he lectured also upon Materia Medica and Chemistry, delivering the Physic and Chemistry lectures annually, until, in 1755, he was appointed to the chair of Chemistry in the University of Edinburgh

When Cullen took up his abode in Glasgow, the medical curriculum of the University was very imperfectly carried out, and it was Cullen's ambition to found at Glasgow a medical school similar to that which had been already established at Edinburgh. To achieve this aim he laboured with characteristic diligence and ardour, until he removed to Edinburgh.

The readiness of apprehension, retentiveness of memory, and energy of disposition which had distinguished Cullen as a boy, characterized him also as a man; but, matured by persistent study, these qualities were manifested by great accuracy of observation, clearness of perception, precision in reasoning, and soundness of judgment. Whether as the medical practitioner, the magistrate, or the professor, he was the same energetic, precise, clear-headed man. As a doctor he early inspired unbounded confidence; as a magistrate he was much looked up to from the minute knowledge he displayed of rural and agricultural affairs, and from the activity he showed in local improvements; and as a professor, his facility of diction, aptitude of description, and above all his admirable method of tuition, quickly secured the admiration of students, and cannot well be surpassed.

At the very outset of his professorial duties he cast aside the effete scholasticism which hung about the academical medical tuition of the period, and addressed himself to the task of conveying in the clearest modes the truths which he had to teach. He laid aside Latin, then the received language for conveying scientific instruction, and adopted the vernacular, delivering his lectures from notes. In the first lecture he gave in the University of Glasgow, he remarked, "written lectures might be more correct in the diction and fluent in the style, but they would have taken up too much time, that may be rendered otherwise useful. I shall be as correct as possible, but perhaps a familiar style will prove more agreeable than a formal one, and the delivery most fitted to command attention."

The great feature of his lectures was the mode in which he laid before the students his own peculiarly careful but sagacious habits of thought. Every stage of the processes by which he had arrived at this or that conclusion was fully set forth, every word clearly defined, every fact or theory methodically arranged; so that each series of lectures constituted not merely a course of tuition in the principles of this or that science, but an invaluable practical illustration of the right mode of thinking. His teaching was free from oracular dogmatism, and consisted mainly in the submission of facts, theories, and opinions to the students the value of which it might rest with them to demonstrate or controvert. Hence the doubtful points were carefully noted, and a method of testing clearly indicated. He, indeed, spoke and wrote as he had thought. His lectures were an appeal to the students' reason rather than their memory. To comprehend the lecturer, it was necessary to think after him, and thus, while the most certain facts of medicine and the collateral sciences were taught by Cullen, he aroused the reflective powers of his hearers, which course, while it raised them in their own estimation, gave birth to a healthy emulation in their studies, and to an attachment towards their teacher which brightens the whole course of his history.

It is impossible to depict Cullen except in his own words, and from the wealth of illustrations contained in his writings and works we shall endeavour to cull sufficient to exhibit his habits of thought.

Let us look upon him first in the chair of Chemistry, the philosophy of which lay almost entirely unexplored when Cullen began to teach. To quote his own story of the position of this science at the time:—

"Chemistry is an art that has furnished the world with a great number of useful facts, and has thereby contributed to the improvement of many arts; but these facts lie scattered in many different books, involved in obscure terms, mixed with many falsehoods, and joined to a great deal of false philosophy; so that it is no great wonder that chemistry has not been so much studied as might have been expected with regard to so useful a branch of knowledge, and that many professors are themselves but very superficially acquainted with it. But it was particularly to be expected, that, since it has been taught in universities, the difficulties in this study should have been in some measure removed; that the art should have been put into form, and a system of it attempted, the scattered facts collected and arranged in a proper order. But this has not yet been done; chemistry has not yet been taught but upon a narrow plan. The teachers of it have still confined themselves to the purposes of pharmacy and medicine, and that comprehends a small branch of chemistry, and even that, by being a single branch, could not by itself be tolerably explained. I do not choose the invidious task of derogating from established reputations, but, were it necessary, I could easily show that the most celebrated attempts towards a system or course of chemistry are extremely incomplete, as examining but a few of the objects of chemistry; that of those examined a very scanty and imperfect account of their relations to other bodies is given, and that even what is given is in a method inconvenient and faulty."

The desire to reduce the facts with which he had to deal to a methodic and comprehensive plan, not only as the most philosophical course, but as the one best facilitating study and research, was one of the most marked of Cullen's habits of thought. It was the reflection, moreover, of a deep belief in the uniformity of the laws governing every portion of the realm of nature, and by his systematic arrangements he sought an approximate expression to these laws. Yet to himself, as to his students, he never for a moment concealed the fact that the method of arrangement which he adopted did not terminate, but simply aided research. Several years subsequent to the delivery of the lecture from which the foregoing paragraph is quoted, he said to his class:—

"After teaching for so many years, it might be supposed that my plan was exactly fixed and sufficiently known: but truly I am yet far from being satisfied with the perfection of my plan, and very certain that it is neither so complete, nor so exactly suited to your purpose as I could wish. It will, therefore, be a long time yet—I hope at least it will be long (for it will only be when the languor and debility of age shall restrain me)—that I shall cease to make some corrections to my plan, some additions to my course."

Equally illustrative of the character of the man is a paragraph immediately following the one we have quoted on the state of chemistry. It is this:—

"From what I have now said, you will judge of the state of chemical learning, and what a difficult task I undertook when I engaged to teach chemistry; and it is very necessary to tell you, that I did not engage in it from any confidence of my abilities, but because it was thought proper to be undertaken, and nobody else was found to do it, and if I can be so lucky as to engage you to apply to the study, I dare say that the more you become acquainted with it, the less will my performance need an apology to you."

The example which Cullen held up to his chemical students for emulation is one which in no small degree applies to himself. He said:—

"If I were now to point out a model for the imitation of the students of chemistry, I could prefer none to this excellent chemist (M. Maargraaf, of Berlin). The curiosity of his researches, his judgment in the choice of experiments, the accuracy of his execution, the perspicuity with which he narrates them, and withal his temperance in theory, are qualities that deserve to be much admired and constantly imitated."

Cullen for several years devoted the whole of his energies to the experimental and philosophical study of chemistry. He succeeded in taking the science out of the hands of artists, metallurgists and pharmaceutists, and exhibiting it as a liberal art, the fit study for a gentleman; and had not his talents been diverted into another course, in all probability his efforts would have been crowned with the richest discoveries. Cullen appears to have been the first who made use of diagrams to illustrate the obscure questions of double elective affinities. It may be noted also that among his pupils was the celebrated Dr. Joseph Black, the discoverer of *latent heat*.

When Cullen began to lecture on medicine, the writings of Boerhaave constituted the chief authority upon the subject in the majority of the medical schools of Europe. This distinguished physician, guided by his profound learning, and by the extensive knowledge he possessed of the then existing state of medicine and the collateral sciences; influenced also by the psychical and vital theories of his distinguished contemporaries, Stahl and Hoffman, had clearly seen the errors of the iatro-chemical and iatro-mathematical schools of biologists, and adopted a system of eclecticism which long formed the basis of medical teaching. He admitted that there were many truths in medical physiology which could only be arrived at by the aid of chemistry; but with Boyle, Hoffman, and others, he contended against the whimsical chemical theories of life and disease which had obtained vogue, and asserted that one of the most valuable uses of chemistry was to expose these pseudo-chemical errors. He contended also against the opinion that the *microcosm*, as well as the *macrocosm*, was governed by mechanical principles. He held that, for the formation of a complete medical physiology, it was requisite that the anatomist should faithfully describe the organism; that the mechanician should apply his particular science to the solids; that by hydrostatics the laws of the fluids in general should be explained, and by hydraulics, their action as they move through the various canals of the body; and, lastly, that the chemist should add to all these, whatever his art, when fairly and carefully applied, had been able to discover; "and then," he remarks, "if I am not mistaken, we shall have a comple account of medical physiology."

While, therefore, this learned teacher had, as it were, selected all that was most worthy from the different physiological systems in vogue, and approximated the necessary elements of a sound system of medicine, he had failed to link them together by any comprehensive idea of the nature of the living body—his physiology lacked life. This defect was early seen by Cullen. He says:—

"When I studied in this university (Edinburgh), about forty-eight years ago, I learned the system of Boerhaave; and, except it may be the names of some ancient writers—of Sydenham, and a few other practical authors, I heard of no other names of writers on physic; and I was taught to think the system of Boerhaave to be very perfect, complete, and sufficient. But when I returned from the university, being very much addicted to study, I soon met with other books that engaged my attention, particularly with Baglivi's specimen *De Fibrâ Motrice et Morbosâ*, and, at length, with the works of Hoffman. Both of these opened my views with respect to the animal economy, and made me perceive something wanting, and to be added to the system of Boerhaave. I prosecuted the inquiry; and, according to the opportunities I had in practice and reading, I cultivated the new ideas I had got, and formed to myself a system in many respects different from my masters. About twenty years after I had left this university, I was again called to it to take a professor's chair, when I still found the system of Boerhaave prevailing as much as ever, and even without any notice taken of what Boerhaave himself, and his commentator, Van Swieten, had in the meantime added to the system. Soon after I came here I was engaged to give clinical—that is, practical lectures; and in these I ventured to give my own opinion of the nature and cure of diseases—different, in several respects, from that of the Boerhaavians. This soon produced an outcry against me. In a public college, as I happened to be a professor of chemistry, I was called a Paracelsus, a Van Hellmont, a whimsical innovator; and great pains were taken in private to disparage myself and my doctrines . . . I truly esteem Dr. Boerhaave as a philosopher, a physician, and the author of a system more perfect than anything that had gone before, and as perfect as the state of science in his time would permit of. But with all this I became more and more confirmed in my own ideas; and especially from hence, that I found my pupils adopted them very readily. I was, however, no violent reformer; and, by degrees only, I ventured to point out the imperfections, and even the errors, of Dr. Boerhaave's system; and I have now done the same in the preface which I have given to the new edition of the First Lines."

Cullen was peculiarly influenced by the doctrines of Hoffman, and subsequently by the physiology of Haller, who had seen that "la physiologie n'etait que l'anatomie animée; *physiologia est animata anatome*;"* and he endeavoured to effect for physiology what he had attempted for chemistry. He sought to grasp those laws which bound together the complex functions of the human organism. In so doing he fashioned into a more comprehensive and simpler system the vast accumulation of facts and opinions under which physiological and medical science still languished; and he stands prominent as one of the great architects, who in successive periods have added another story to the slowy erected structure of scientific medicine.

The point of chief moment in Cullen's physiology, was the importance which he attached to the nervous system in the animal economy. He believed that upon the functions of this system "life depends, and that its energy, therefore, is necessary to the whole." His contemporaries, Haller, Whytt, and Gaubius had already beaten this track, but probing their notions, as it were, with the more abstract ideas of Hoffman, Cullen methodized, improved, and extended the ideas entertained of the influence of the nervous system in the animal economy. This was an important step in advance, and gave an immense impulse to the philosophical study of practical medicine in this country; for upon his conceptions of the influence which the nervous system exerts upon the organism, were mainly founded the opinions which Cullen taught concerning the causes of disease and the operation of medicines.

It is sufficient that we should simply note the direction which the growth of medical science took under Cullen's hands, as indicating his habits of thought, without pursuing the subject with any degree of minuteness. We have to deal with those points which may serve to show the fundamental tendencies of his intellect.

* Blainville.

Now Hoffman was the most distinguished of those biologists who have been classed by Whewell as the vital-fluid school. They held that the whole of the peculiar functions of *life* depended upon the agency of a subtle ethereal substance diffused throughout the frame. The iatro-mathematical school had left unsolved an essential question in the phenomena of organic life. Motion being given, it was possible to construct on mathematical principles a scheme of its modifications and transference. But in the living organism there was manifested a spontaneous commencement of motion, which was not explicable by any mechanical action. Hoffman attempted to solve this difficulty by the assumption of a principle higher in kind than that of mechanical force. He held that the Ether, a hypothetical material agent of extreme subtilty and energy, supposed to permeate all nature, was the active agent by which was brought about the whole of the phenomena of organic life. He conceived that in animals this agent was separated from the blood, and stored in the brain, from which, by means of the nerves, its energizing power was extended to the whole body.

The influence of Hoffman's views upon Cullen appears to have been in directing his attention markedly to the nervous system as the great source of animal energy and power. Gaubius' philosophical views on the reciprocal influence of the mind and body upon each other, Haller's profound researches upon "irritability," and Whytt's ingenious speculations and experiments upon vital and other involuntary motions, and his observations upon the connexion of sympathetic movements with the nervous system, as well as the scattered opinions and inquiries of other physiologists, all contributed to support Cullen's conception; and in this matrix he developed those views of the nervous system which play so essential a part in his physiological and pathological writings. He bound together the scattered physiology of the period into a connected and coherent system, and this with a philosophical acumen and depth of thought that has hardly yet been appreciated.

Rejecting the notions which Hoffman attached to his hypothesis of a vital fluid, and of the mode in which it was distributed in the body, he nevertheless adopted the phrase *nervous fluid* to express the capacity possessed by the nervous system to transmit impressions. "By nervous fluid," he says, "I mean nothing more than that there is a condition of nerves which fits them for the communication of motion."

It is difficult to conceive a more inapt term to convey so clear and philosophical an expression of the nature of nervous action. The one devoid of theory, eminently calculated to facilitate inquiry, and far in advance of the physiological speculations of the period; the other conveying the notion of a medium of action absolutely hypothetical, and yet in its nature such as to give a certain definite familiar notion to every one, and to furnish a seemingly ready explanation of an abstruse subject. That the term has been often retained by the followers of Cullen, while the signification he attached to it has been lost, and that his doctrines have at times suffered from this cause, is, we think, certain.

No more striking illustration of this source of error in Cullen's teaching, and at the same time no better example of his careful distinction of theory from fact, can be given than in his definition of the words *excitement* and *collapse*, the former of which in the hands of one of his disciples became the exponent of one of the boldest and most unscrupulous theories that ever disturbed medicine. Cullen writes:—

"I take it for granted that when you consider the weakness and manifest mistakes of any other hypothesis, you will readily, with me, think that the condition of the nerves fitting them for the communication of motion, consists in some state of the matter of the nervous fluid itself, and of its having more or less mobility, in some cases being capable of being moved with more ease and vigour, while in other cases it is unfit for either. Now, I say, merely to avoid long expressions, I shall choose shorter ones, and shall speak of the moveable state of the nervous fluid, or of that condition of the nervous system which fits it for the communication of motion, under the term of its *excitement*, and a deficiency or lesser degree of this, I shall call *collapse*. Now, you must merely consider these as terms employed for what I take to be matters of fact, the increased or diminished force of the animal power or energy of the brain, and not as imparting matter of theory, or as expressing anything with regard to the action of the nervous fluid, or wherein these different states of the nervous system consist. Whatever hypothesis I may have fancied to myself, I consider these as hypotheses still, and dare not trust you with them, unless you take them as they pass in my mind, and be very certain never to apply them in particular cases."

Elsewhere Cullen has remarked, "My business is not so much to explain how this and that happens, as to examine what is truly matter of fact." "My anxiety is not so much to find out *how* it happens, as to find out *what* happens." This, indeed, constituted a chief feature of his intellectual character and teaching, and it is evinced in the carefulness with which he defines the signification which he attaches to the words he makes use of, as in the instances cited. But it unfortunately happened that the terms mentioned necessarily involved in their mode of use a theoretical form of expression, if not a theory, and the onward impulse given to medicine by Cullen was quickly clouded in no small degree by theories based upon these very terms. No more instructive illustration could be advanced of the evils arising from the introduction of common words into science as technical terms, where the meaning cannot be fixed without ambiguity.

The same remarks are also applicable to the use which Cullen made of the term *spasm*, to express the state of the vascular system in the different stages of fever. The word had been long a common one in medicine, and was familiarly employed to express certain well-known phenomena. Hence the precise conception which Cullen wished to attach to it was exposed to the chance of being jumbled up into a confused meaning, with every individual's peculiar apprehension of the import of the word. Cullen's views of the nature of fever were singularly enlightened and advanced, but the word adverted to has been an unfailing theme of contention and misapprehension from his own to the present time. Read his description of fever without the use of the word, and we must admit that we can add but little to the general expression. He says:—

"I suppose that in every fever there is a power applied to the body which has a tendency to hurt and destroy it, and which produces in it certain motions which deviate from the natural state, and at the same time, in every fever which has its full course, I suppose that, in consequence of the constitution of the animal economy, there are certain motions excited which have a tendency to obviate the effects of the noxious powers, or to correct or remove them. Both these kinds of motion are considered as constituting the disease. But the former is, perhaps, strictly the morbid state, while the latter is to be considered as the operation of the *vis medicatrix naturæ*, of salutary tendency, and which I shall hereafter call the REACTION of the system.

"In what manner the state of debility brings on, with respect to the heart and arteries, a seemingly contrary state of force and activity, is a difficulty which we do not pretend entirely to remove. We can only refer it to a general law of the system, which, however, is very well established, and is shortly this:—That where a deviation from the natural state of health happens, from the nature of the economy this deviation naturally produces a tendency in the system to restore itself to its former condition. This, I say, constitutes the *vis preservatrix* and *medicatrix naturæ*."

And yet no man was more alive than Cullen to the necessity of the accurate use of terms. Explaining the reason why he entered very fully into the meaning which should be attached to the word symptom, he remarked:—

"The use of such generalities will not be immediately obvious, but some accuracy in your ideas and language with regard to them, you will find on many occasions to be of consequence. It is proper that you should be sometimes exercised in forming abstract and general notions. Without these you may think of acquiring a greater number of particular facts; but till the terms in which these particular facts are expressed are accurately defined, and till the facts themselves are digested into a system, they will not be very useful or even of much practical application."

Cullen's views regarding the nervous system as the connecting medium between the immaterial and material parts of man were far-sighted and profound. He considered that mental changes and certain physical alterations in the nervous system were interdependent; he says:—

"Although the soul is certainly a distinct substance from the body, it appears highly probable that the soul, while connected with the body, seldom acts, but in consequence of motions first excited in the latter. . . . The soul is, indeed, a necessary part of these motions, but it cannot be said to direct and govern them independently of the conditions of the body. . . . Whatever share the soul may have in supporting the motions of the body, we can have no art of physic but in so far as we suppose that the causes operating upon the body act by a physical necessity, and that by a knowledge of such causes we can produce certain changes in the state of the matter and organization of the body."

The opinions with which Cullen set out in the formation of his celebrated Nosology were eminently philosophical, and well calculated to illustrate his method of thinking. In a clinical lecture he delivered the year previous to the publication of his first edition, he said, after reviewing the defects in the nosologies then existing:—

"These are the faults in the systems hitherto given. I must offer you another. The matter is so difficult that I have hesitated much about making an attempt; but I hazard something to be useful, and an imperfect attempt is allowable here, though not fit to be offered to the public. As it is now given out, it is to be regarded as a sketch not finished."

He aimed at simplifying the existing systems of Sauvages and others, and giving more accurate descriptions of the principal genera of diseases. In the preface to the second edition of the Nosology, he stated that probably as long a period would intervene between the publication of Sauvages' nosology and a good system, as did between the first attempt at botanical arrangement by Cæsalpinus and the writings of Linnæus, adding that he aspired to the humble merit of exciting others to the study of his favourite science.

In no respect were Cullen's remarkable practical tact and philosophical sagacity brought more effectually into play than in the reformation of the Materia Medica. Previous to the time when Cullen's influence as a teacher of Materia Medica became apparent, although many preparations belonging to mystical medicine had been excluded from the London Pharmacopœia, and several from the Edinburgh, both works still retained much of the character of Dr. Hornbook's pharmaceutical repertory.

"Calces o' fossils, earth, and trees;
True Sal-marinum o' the seas;
The Farina of beans and pease,
He has't in plenty;
Aqua-fontis, what you please,
He can content ye.

Forbye some new, uncommon weapons,
Urinus Spiritus of capons;
Or Mite-horn shavings, filings, scrapings,
Distill'd *per se;*
Sal-alkali o' Midge-tail-clippings,
And monie mae."

Thus, along with a redundancy of vegetable samples, the pharmacopœias of both countries contained among the animal substances, the fat of the goose, duck and swan, cobweb, the entire toad, the spawn of the frog, ants entire, and ants' eggs, the human skull and mummy, snails, earthworms, millipeds, the excrement of the dog, scorpion, viper, &c. Among the compounds were the Mithridate or Confection of Damocrates, consisting of forty-seven ingredients with a proportion of Canary wine, and the Theriac of Andromachus, consisting of sixty-four ingredients with Canary wine. In the fifth edition of the Edinburgh Pharmacopœia many of these things were excluded, and in the sixth, chiefly through the influence of Cullen, the work was almost entirely depurated. Of the preparations and animal substances named, millipeds only were retained. The London College had been the first to commence the reform of the Pharmacopœia, but the Edinburgh first achieved a complete reform. Cullen, also, greatly curtailed the list of vegetable simples in the Pharmacopœia.

The reforms briefly glanced at, however, were the necessary result of Cullen's essentially practical but highly philosophical method of teaching Materia Medica and Therapeutics. The former he reduced to a system which occupies a very important position in the history of medical science, and the latter he taught in its widest scope and truest form. The term therapeutics had previous to his time been almost solely confined to the study of those general principles which should guide us in the administration of remedies as means of curing diseases, while the study of the means of preventing diseases had been supposed to constitute another branch of medicine—Hygiene.

Cullen, however, rightly held, that "Hygiene and Therapeutics must form one and the same general doctrine."

As a clinical teacher Cullen was eminently practical, taking advantage of every circumstance that could awaken the observation of the students, and drawing particular attention to diseases of ordinary occurrence, which must form the groundwork of all sound medical experience. He taught also from the commencement of lecturing the great advantages of simplicity in prescribing, a rare novelty in those days of bewildering compounds. He showed how simplicity was necessary for the right appreciation of the effects of such drugs as were administered, and to secure their legitimate operation.

In everything that Cullen directed his attention to, he displayed the same untiring energy, activity of thought, and comprehensive method of dealing with, methodizing, and extending existing information. He sought to clear away the redundancy of theory which hampered the writings of the great authorities in medicine and the collateral sciences, and to reduce these to a system founded chiefly on observation and experience, and thus place them on a firmer foundation for subsequent research and advancement. This high and philosophical aim he achieved. He never lost sight himself of the true character of his researches, and he terms his most important attempts at generalization simply "approximations to truth." No man, moreover, ever exhibited a juster medium between philosophical speculation and practical application. It is one of the necessary consequences of medical science that an immediate practical end must almost invariably be kept in view. Physicians seek that they may relieve, and they are always liable (to use the language of Bacon) to—

"Turn with premature and hasty zeal to practice, not merely on account of the advantage to be derived from it, but in order to seize upon some security in a new undertaking of their not employing the remainder of their labour unprofitably, and by making themselves conspicuous, to acquire a greater name for the pursuit. Hence, like Atalanta, they leave the course to pick up the golden apple, interrupting their speed, and giving up the victory."*

Cullen never lost sight of the great object of medical science, but he so tempered his practice with speculation, and his speculation with his practice, that each became the legitimate helper of the other, and they advanced with equal steps.

It is instructive to note the varied manner in which Cullen employed his talents, in addition to those labours upon which his fame rests. He had an accurate and critical knowledge of

* *Novum Organum*, Aph. 70.

botany; he thought much and deeply upon agriculture, and of the application of chemistry to it; and in December, 1749, we find Lord Kames writing to him, "My sedate purpose is, that your name shall be carried down to posterity by a treatise on agriculture better than the world ever yet saw." He wrote upon "the mechanical principles on which ploughs have been constructed, to find out what is the importance and effect of each part, and to examine what variation each or all of them require, according to the difference of soil in which they are employed." Alluding to this essay, Lord Kames remarked, in a letter to Cullen, "a thought has struck me in the head within these few days relative to *your engine*, the plough;" and an intelligent farmer who submitted to Cullen a proposition to substitute iron for wood in the construction of ploughs, observes that Cullen was "the first person in Scotland who has attempted to account for the structure of that machine upon mathematical principles." Cullen also suggested certain important improvements in bleaching, and projected a process for the purification of salt.

Among the most notable features of Cullen's mental habits were his equanimity of disposition, and his rare power of winning the affection of those with whom he was thrown most immediately into contact. Notwithstanding the opposition with which his views were at first received by his fellow professors in Edinburgh, he never recriminated, however painful the assault upon him might have been. The interest he took in his pupils was unwearied. He delighted to encourage their efforts and remove their doubts, and this begat a mutual confidence which was marked on the professor's side by genuine kind-heartedness, and on the student's by affectionate respect. The friendship which arose between Cullen and his more emulous and distinguished students endured until death, and the correspondence which passed between him and them after they had been launched into active life, is most pleasing, in the kindly and affectionate interest displayed on both sides. "Dear Joe," and "Dear Willie," are the terms in which he commonly addresses his celebrated pupils Joseph Black and William Hunter, and in the same familiar phrase he wrote to other pupils. In a letter to Dr. Black, Cullen writes:—

"I received your packet of chemistry, which rejoiced me extremely. A new experiment gives me new life; but I wonder at the reserve and ceremony you use with respect to me. Did I learn chemistry from you, only to be a bar to your inquiries? The subject is not so limited as to be easily exhausted, and your experiments will only advance me so much farther on."

On the other hand, when it became probable that the chair of

Chemistry at Edinburgh would become vacant, Dr. Black at once communicated the fact to Cullen, adding :—

"From some hints I have received, I have reason to suspect that I am not excluded the possibility of an offer; but I assure you, doctor, I am absolutely resolved to refuse it, if there is any hopes of its being of any advantage to you."

In 1745, William Hunter writes:—

"Dear doctor,—I could not let slip the opportunity of thanking you for your kind letter; and in answer, I wish I could say anything that might convince you of my sense of your goodness in everything, but particularly in this case. You have interested yourself warmly in my brother's (James's) health; and in the hurry of business and lecture-reading, have taken time to write to me of him. Indeed, it was kind; I thank you in his name, and I thank you for myself. You see I pretend to gratitude; so love me, God, as I am sincere. . . . Well, how does the animal economy appear to you, now that you have examined it, as one may say, with precision? I have good reason to put the question to you, because, in my little attempts that way since I began to think for myself, nature, where I am best disposed to mark her, beams so strong upon me, that I am lost in wonder, and count it sacrilege to measure her meanest feature by my largest conception. Ay, ay, the time will come when our great philosophers will blush to find, that they have talked with as little real knowledge, and as peremptorily of the animal powers, as the country miller who balances the power of Europe."

Then we have Cullen writing to William Hunter:—

"I value your friendship in the highest degree, and owe so much to it that I think you have the best title to advise me—direct me with regard to my sons. I sent them to London in high expectation of the benefits to be received from your friendship and instruction, and I had some confidence in their conduct, else I had not trusted them in London; but in the last article I have the highest satisfaction in finding that they have got your approbation, who are so good a judge, who never did a foolish thing yourself, and therefore may be a little rigorous with respect to others."

We find Dr. Cullen writing to other pupils:—

"Dear Fordyce,—Your letter last night was very welcome. I suspected that your laziness had banished you from me for ever. I won't tell you with what regret I felt that; but the sight of your hand was very agreeable. . . . I shall be glad to see your specimens, and more so to see yourself, when you have been exotic."

Again:—

"Dear Fourie [Balfour Russell],—I shall always be glad to have your letters, when they inform me of your own success and happiness, though they tell me of nothing else. . . . I would fain play off Fordyce and you against each other. Fordyce set out with as handsome a margin as you gave me, but he has very discreetly filled it afterwards. I expect no margins from you hereafter, especially after so long a letter as this is. I mean to show you that one may write a long letter to a friend without saying anything. I may perhaps show you that I can send a letter to another quarter of the globe with no more in it than what I would say in yawning at my own fireside."

As with his pupils so was Cullen with his patients. Dugald Stewart, when a boy of fourteen or fifteen, was attended by Cullen for a slight indisposition and was recommended by him to relax his studies, and have recourse to some light reading, *Don Quixote* being suggested :—

"In subsequent visits to this patient," Dr. John Thomson states, "Cullen never failed to examine him in the progress he had made in reading the humorous story of the great pattern of chivalry, and to talk over with him every successive incident, scene, and character in that history. In mentioning these particulars, Mr. Stewart remarked that he could never look back on that intercourse without feeling surprise at the minute accuracy with which Cullen remembered every passage in the life of Don Quixote, and the lively manner in which he sympathized with him in the pleasure he derived from the first perusal of that entertaining romance."

Of Cullen's family habits of thought, a charming illustration is preserved in a letter addressed to one of his sons, then leaving home for the first time. As we read this delightful epistle the inmost peculiarities of the man's thoughts appear to be opened out before us.

"*Edinburgh*, 11*th November*, 1765.

"Dear Jamie,—I am afraid you do not consider the anxiety we have about you, or you would have continued to give us a letter from Glasgow ; but I hope you will mind better for the future to take every opportunity of giving us accounts of you. We have received your letter to-day, and Mr. Leitch and your brother Patrick came home before dinner. Your letter is a little concise, and I beg you will be always as full as you can, for we wish to know every particular that happens to you. Keep memorandums of what you may write; so that, when you sit down, your materials may be ready. It is common in sea journals to set down occurrences, though not relating to the reckoning. I wish you would mark as many as you can, if not in your journal, at least in another book. One cannot begin too early to make remarks on everything. It is a very improving exercise, and through life, attention and observation are the foundation of success, and distinguish the able and wise from the weak and foolish.

"I hope you have got everything necessary, and a proper chest to put them in. I beg you will study to keep it always in good order, and all your clothes in good condition ; and, particularly, I expect to observe that while you have perused your books you have also kept them clean. As I hear that Mr. Kippen is not well, if he is not able to go with you, it will pretty certainly take you into the store at Antigua, which I shall think very lucky; but I tremble for your handwriting, and I beg of you, in the most earnest manner, to take pains on that article. If you have any regard to my satisfaction you will; and, for your own sake, consider that nothing so much gives the appearance of mean and low breed as bad writing. Take every opportunity, therefore, to practise with attention, and, if possible, never without it. As you are not to go to Ireland, you may find the two dissertations an agreeable present to some doctors in the West Indies. I find Mr. Anderson refuses to take your money, but press him to it again, and at least to keep it to lay out for you. If he declines it in every shape, I desire you may leave it with Mr. Hamilton, as I have much use for it. In the meantime, let me know what he lays out for you. Dear Jamie, I hope to write to you, and, perhaps, to send you letters for Virginia. I shall be glad to know what letters you get from your uncle. I hope few advices are now necessary. Study your trade eagerly, decline no labour, recommend yourself by briskness and diligence, bear hardships with patience and resolution, be obliging to everybody, whether above or below you, and hold up your head both in a literal and figurative sense. I trust that honour, truth, and discretion, shall always guide you, and give the utmost comfort to, my dear Jamie, your affectionate father,

"WILLIAM CULLEN."

It is requisite to note that Cullen's kind-heartedness rendered him indifferent to pecuniary success, and when he died he left to his family little else than his great fame.

Among the intimate acquaintances of Cullen were David Hume, Adam Smith, Lord Kames, and Robertson the historian, and there is little doubt that the writings and companionship of these gifted men exercised no small influence upon Cullen's tone of thought. Contemporary with him, in the medical profession, were Huxham, George Cleghorn, Sir John Pringle, Donald Monro, Francis Home, Brocklesby, the two Linds, and Sims—all men who laboured to establish medicine upon observation and experience alone, and whose writings contributed rich material for the active, generalizing mind of Cullen. He had, as his biographer remarks, "fallen on good days;" but these days were necessary for the development of a Cullen. The most powerful genius would abort unless the circumstances necessary to its development were present. The great man may be said to be as much the product of the epoch in which he lives as of his own intellectual greatness. Both elements are necessary for the full birth of mental grandeur. When Cullen began to study, theoretical medicine had commenced to wane, and the sciences of organization and medicine were fast becoming matters of true observation and experiment. Many inquirers were in the field, and facts and observations accumulated rapidly; but both medicine and the collateral sciences lacked connexion and coherency. Cullen's sagacity early saw this, and he set himself steadily to work to remedy the evil in so far as medicine and the sciences most immediately connected with it were concerned, and in this endeavour he succeeded. He was eminently qualified for the task by his untiring perseverance, accurate observation, cautious, but sound judgment, and intimate practical and experimental acquaintance with the subjects to which he chiefly devoted his care. He linked together the floating facts of the different sciences, and consolidated them into systems in which there was only so much theory as to give a healthy vitality to them. Thus he gave a vast impulse to, and built a sound foundation for, the future study of medicine; and the subsequent history of the science shows how deeply we are indebted to Cullen for its vigorous growth since the period when he taught.

Cullen died on the fifth day of February, 1790, aged seventy-nine years and eleven months. The story of his mental life is one of rare interest. His early cultivation of habits of independent thought, and the never-tiring assiduity he displayed throughout life, show that he won his way to fame by the old, old path of hard, relentless work. Hence the story is one that appeals strongly to the true student. We have assayed to tell somewhat of its character, but he who would rightly know it must turn to the invaluable volumes from which we have derived our knowledge.

---

